# Chinese Americans’ Priorities and Practices for Designating a Healthcare Proxy

**DOI:** 10.1093/geroni/igaa057.1344

**Published:** 2020-12-16

**Authors:** Mandong Liu, Iris Chi

**Affiliations:** University of Southern California, Los Angeles, California, United States

## Abstract

Many Americans need decision-making from someone else due to cognitive impairment. Chinese comprises the largest Asian group in the U.S.; few studies have investigated factors influencing Chinese Americans’ health care proxy (HCP) designation. The aim of this study was to examine factors associated with Chinese Americans’ HCP designation, using Andersen’s Behavioral Model as a lens. Data were from the 2015 Asian American Quality of Life Survey. Hierarchical logistic regression analyses were conducted to test the incremental predictive power of predisposing, enabling, and needs factors. We found that age, marital status, religious affiliation, health insurance, acculturation, and self-rated health were predictors for HCP designation. The odds of having an HCP designation were 1.96 times higher for those aged 65 and above, compared to those aged 18-64 (p=0.045); The odds were 2.40 times higher for those who were married (p=0.006); The odds were 1.79 times higher for Protestants (p=0.042) and 2.25 times higher for Buddhists (p=0.025), compared to those with no religious affiliation; Having a health insurance increased the odds of having an HCP designation by 2.23 (p=0.022); For each additional unit in acculturation score, the odds of having an HCP designation increased by 0.65 (p=0.011); Compared to those who rated their health as excellent/very good/good, those rating health as fair/poor had a 2.97 times higher odds of HCP designation (p=0.001). We concluded that various factors influence Chinese Americans’ HCP designation, and appropriate and innovative practices should be used to assist HCP discussion and designation among Chinese and other ethnic minority populations.

